# Assessment of Electrospun Pellethane-Based Scaffolds for Vascular Tissue Engineering

**DOI:** 10.3390/ma14133678

**Published:** 2021-07-01

**Authors:** Vera Chernonosova, Alexandr Gostev, Ivan Murashov, Boris Chelobanov, Andrey Karpenko, Pavel Laktionov

**Affiliations:** 1 Institute of Chemical Biology and Fundamental Medicine, Siberian Branch, Russian Academy of Sciences, 630090 Novosibirsk, Russia; boris.p.chelobanov@gmail.com (B.C.); lakt@niboch.nsc.ru (P.L.); 2 Meshalkin National Medical Research Center, Ministry of Health of the Russian Federation, 630055 Novosibirsk, Russia; dr.gostev@gmail.com (A.G.); ivmurashov@gmail.com (I.M.); andreikarpenko@rambler.ru (A.K.)

**Keywords:** small-diameter vascular graft, electrospinning, polyurethane Pellethane 2363-80A, endothelialization, functioning in vivo

## Abstract

We examined the physicochemical properties and the biocompatibility and hemocompatibility of electrospun 3D matrices produced using polyurethane Pellethane 2363-80A (Pel-80A) blends Pel-80A with gelatin or/and bivalirudin. Two layers of vascular grafts of 1.8 mm in diameter were manufactured and studied for hemocompatibility ex vivo and functioning in the infrarenal position of Wistar rat abdominal aorta in vivo (*n* = 18). Expanded polytetrafluoroethylene (ePTFE) vascular grafts of similar diameter were implanted as a control (*n* = 18). Scaffolds produced from Pel-80A with Gel showed high stiffness with a long proportional limit and limited influence of wetting on mechanical characteristics. The electrospun matrices with gelatin have moderate capacity to support cell adhesion and proliferation (~30–47%), whereas vascular grafts with bivalirudin in the inner layer have good hemocompatibility ex vivo. The introduction of bivalirudin into grafts inhibited platelet adhesion and does not lead to a change hemolysis and D-dimers concentration. Study in vivo indicates the advantages of Pel-80A grafts over ePTFE in terms of graft occlusion, calcification level, and blood velocity after 6 months of implantation. The thickness of neointima in Pel-80A–based grafts stabilizes after three months (41.84 ± 20.21 µm) and does not increase until six months, demonstrating potential for long-term functioning without stenosis and as a suitable candidate for subsequent preclinical studies in large animals.

## 1. Introduction

Cardiovascular diseases are the leading cause of disability and mortality internationally [1]. Trans-catheter treatment of vascular diseases, like stenting and balloon plastics, greatly improves cardiovascular patient care; however, almost 20% of patients require surgical replacement due to advanced diseases [2,3]. Currently used vascular prostheses (either synthetic and autologous) do not provide long-term functioning of small-diameter vascular grafts (SDVGs) with a diameter <6 mm because they are prone to thrombosis, stenosis, or aneurysmal dilatation [4,5,6]. In accordance with modern concepts, SDVGs (including those produced from synthetic polymers) should have biomechanical properties similar to a natural vessel (compliance with natural artery, durability in pulsatile flow) and a surface supporting effective endothelialization; these factors are essential for the long-term functioning of SDVGs [6,7]. Considering the demands of the medical market [8], the development of SDVGs is an urgent task in tissue engineering.

Among different methods of SDVG production, electrospinning deservedly occupies an exclusive position [7,9,10]. This method is simple and inexpensive to implement and manufactures porous structures consisting of nanofibers similar to the native extracellular matrix [10,11]. This method produces SDVGs from natural polymers, as well as various synthetic polymers and their mixtures with natural polymers [10,12,13].

Of the synthetic polymers used for SDVG production, polyurethanes (PUs) can be highlighted due to their strength elasticity and good biocompatibility and hemocompatibility. In medicine, they are widely used for the manufacturing of biomedical products, including those in long-term contact with blood, such as blood bags, vascular catheters, artificial heart diaphragms, artificial hearts, arteriovenous shunts, and heart valves [14,15,16,17]. Aliphatic or aromatic diisocyanates are the basic reagents in the synthesis of PUs [18]. Polyurethanes synthesized from the aromatic diisocyanate are less hydrophilic, have a significantly higher tensile strength being wetted, and, as a rule, have higher fatigue strength compared to PU based on aliphatic diisocyanate. The biocompatibility of aromatic PU materials is generally lower than that of aliphatic PU products, but can be increased using various approaches [17,19]. In particular, the blending of PU with natural polymers (gelatin, collagen, chitosan, etc.) in the electrospinning solution produces novel materials, demonstrating increased cell adhesion and proliferation on their surface, as well as improved mechanical characteristics compared to the initial PU [20,21,22,23,24].

Pellethane 2363-80A is a kind of thermoplastic elastomer with an aromatic hard segment. When produced by Lubrizol Advanced Materials, it is approved for production of medical devices (https://www.lubrizol.com/Our-Company/Live-Better (accessed on 7 May 2021)). In this study, we investigated materials fabricated from this polyurethane through in vivo and in vitro experiments. We examined the effects of gelatin or/and bivalirudin supplemented into a PU solution for electrospinning on the structure and properties of 3D matrices and their biocompatibility and hemocompatibility. Materials demonstrating the best characteristics were used to manufacture SDVGs and study their functioning in the infrarenal position of the abdominal rat aorta.

## 2. Materials and Methods

### 2.1. Electrospinning of Matrices

The solutions for electrospinning were prepared using polymer (Pellethane 2363-80A (Pel-80A), Lubrizol Advanced Materials, Ritterhude, Germany), proteins (gelatin (Gel) and bivalirudin (Bv), Sigma, Missouri, MO, USA) and 1,1,1,3,3,3-hexafluoroisopropanol as a solvent in accordance to earlier described protocols [25]. To determine biocompatibility and hemocompatibility ex vivo, in vivo functioning electrospun scaffolds were produced using solutions of 3.5% Pel-80A, 3.5% Pel-80A + 10% Gel, 3.5% Pel-80A + 1.5% Bv or 3.5% Pel-80A + 10% Gel + 1.5% Bv.

The matrices (thickness = 150–160 µm) and vascular grafts (1.8 mm diameter, wall thickness = 130–150 µm) were fabricated using an NF-103 (MECC, Ogori-shi, Japan) electrospinning device under the following conditions: the feed rate 1–1.15 mL/h, capillary-collector distance 19–20 cm, voltage 18.5–24 kV, and rotation speed of drum collector 300 rpm.

The electrospun scaffolds were evacuated under forvacuum for 12 h to evaporate remaining solvent, packed with zip-lock polyethylene containers, sterilized with 25 kGy electron beam irradiation using an ILU-6 accelerator (Institute of Nuclear Physics, SB RAS), and stored at 4 °C.

### 2.2. Characterization of Electrospun Scaffolds

Scanning electron microscopy (SEM) was used to test surfaces of the materials, including fibers diameter and pore sizes, as earlier described [23]. The contact angle was determined with a Drop Shape Analyzer DSA 25 (Kruss GmbH, Hamburg, Germany), as described in the manufacturer’s instructions.

A Universal Zwick/Roell Z100 (Zwick Roell AG, Ulm, Germany) test bench was used to study mechanical properties of scaffolds, as described in ISO 7198:1998 (4 replicates for every scaffold) [23]. A “wet” sample was obtained after incubation of a matrix in water for 30 min at 20–23 °C to moisten the 3D matrix completely. The strength and elongation of matrices were determined from the constructed tensile curves in four replicates. Young’s modulus was determined in experiments with statistical loading. Before testing, the material was conditioned by loading the test specimen 5 times (each for 10–20 s) using 30–50% of the elastic limit load determined in the preliminary test. The static load was increased in steps of 10 g (to 60–70% of the elastic limit) that were applied for 20 s with a subsequent measuring of the deformation after unloading. The load that induced more than 5% elongation after unloading was considered the elastic limit.

### 2.3. Interaction of Endothelial Cells on 3D Matrices

#### 2.3.1. Cell Viability Assay

The scaffolds (discs with a diameter of 10 mm) were pre-incubated with the culture medium for 2 h to achieve complete moistening of the material. Human umbilical vein endothelial cells (HUVEC) were isolated from umbilical vein of newborn by collagenase IV. Culture medium was removed from the wells before seeding the HUVEC cells on the scaffolds (2000–8000 cells per well). The seeding density of cells was about 50–65% of the monolayer. Control cells were cultivated in parallel on tissue culture polystyrene. The viability of endothelial cells was assessed after 48-h cultivation on the surface of the matrices. The culture medium was removed and the cells were incubated with Alamar Blue^®^ (Invitrogen, Eugene, OR, USA), as earlier described [26]. Matrices without cells were used as a control for assessing the dye sorption on the tested materials.

#### 2.3.2. SEM and Fluorescent Microscopy of Matrices with HUVEC

The matrices with HUVEC after 48 h incubation were washed twice with phosphate buffer to remove non-adherent cells and fixed in 2% formaldehyde in a phosphate buffer for 30 min. Graded ethanol series (50%, 70%, 90%, and 100%) were used for sample dehydration before incubation in a mixture of hexamethyldisilazane (HMDS) with ethanol (1:1), and then 100% HMDS in order to prepare 3D matrices with cells for study by a EVO 10 (Carl Zeiss AG, Jena, Germany) scanning electron microscope.

HUVEC cells cultivated at 3D matrices during 48 h were fixed in 2% formaldehyde in a phosphate buffer and stained with Hoechst 33342 (Invitrogen, USA) and Phalloidin-TRITC (Sigma, USA) for 20 min at 37 °C for subsequent fluorescent microscopy. For this study, we used a confocal laser scanning microscope LSM 780 NLO (Carl Zeiss, Jena, Germany) at 405 nm to detect cell nuclei and at 543 nm to detect actin microfilaments with ZEN 2 software (Carl Zeiss Inc., München, Germany).

### 2.4. Hemocompatibility of 3D Matrices

#### 2.4.1. Hemolysis Test

The detailed protocol of this assay was outlined in a previous manuscript [26]. Tests were performed in three replicates for all types of materials.

#### 2.4.2. Assessment of Hemostasis Parameters after Contact of Blood with Tubes

Fresh donor blood was pumped through the tubular graft with an inner diameter of 1.8 mm using a custom-made test bench consisting of two syringes moving synchronously while pumping blood through a vascular graft (VG) located between them for 30 min at 24 mL/min. The concentration of D-dimers and activated partial thromboplastin time (APTT) was measured using kits from Siemens Healthcare Diagnostics Products GmbH (Marburg, Germany) against untreated blood. Tests were performed in three replicates for all types of vascular grafts.

For SEM, samples after interaction to blood were prepared as described above for materials with endothelial cells.

### 2.5. Graft Implantation

Anesthesia and implantation procedures followed an earlier described protocol [27]. Briefly, we used 18 Wistar rats with an average age of 6.8 ± 0.15 months and average bodyweight of 440 ± 40 g. The animals were kept under stationary vivarium conditions with natural illumination, standard diet, and water ad libitum. The 18 e-PTFE grafts without coating (inner diameter = 1.8 mm, 120 µm wall thickness; Ekoflon, Russia) were used as a control group. Implantation of experimental and control SDVG was done simultaneously in the same experimental set, while animals were randomized between groups. The electrospun grafts (outer layer from 3.5% Pel-80A + 10% Gel + 1.5% and inner layer from 3.5% Pel-80A + 10% Gel + 1.5% Bv (thickness of 10–20 µm)) of length 10 ± 2 mm were implanted to the infrarenal aorta of six animals and studied after the observation period (1 week, 3 and 6 months).

### 2.6. In vivo Monitoring and Graft Explantation

An ultrasound examination was performed for all 6 animals of the group at 1 week, 3 and 6 months by a DC7 ultrasound system (Mindray, Shenzhen, China). The average linear blood flow velocity in the VG was estimated in different regions before the proximal and after the distal anastomoses between the aorta and VG, which is the middle portion of VG.

The specimens, including 5 mm of the aorta adjacent to the anastomoses, were excised immediately after an ultrasound test with subsequent rat euthanasia. Explanted VGs were rinsed with phosphate buffer to remove blood cells, fixed in 4% formaldehyde, and examined under a SteREO Discovery V12 (Carl Zeiss, Munchen, Germany) microscope measuring outer and inner diameters, and the thickness of neointima.

### 2.7. Histological and Immunohistochemical Examination of Grafts

Cross-sections of VGs (thickness of 10 µm) were obtained using a Microm HM-550 cryomicrotome (Carl Zeiss, Germany) and used for histological and immunohistochemical examination. Samples were stained with hematoxylin and eosin following a classic protocol (H&E; Biovitrum, St. Petersburg, Russia). A detailed protocol of immunohistochemical staining by primary monoclonal and polyclonal antibodies of type IV collagen (clone PHM-12 + CIV22), α-SMA (clone 1A4/asm-1), and factor VIII (rabbit polyclonal) conjugated with horseradish peroxidase was outlined in manuscript [26].

The thickness of neointima and calcified area were assessed quantitatively as previously described [27]. Calcified regions were determined as dark purple regions on H&E sections, as described in manuscript [28].

### 2.8. Statistical Analysis

The data were accumulated and initially sorted using Microsoft Excel 2010. The Statistica 10 software package in Windows 7 (StatSoft Inc., Tulsa, OK, USA) was used for statistical data processing.

### 2.9. Ethical Statement

Manipulations with animals complied with the European Convention on the Protection of Laboratory Animals [29]. The study was approved by the ethical committee of ICBFM SB RAS.

## 3. Results

### 3.1. Fabrication and Characterization of Scaffolds

As previously described, Pel-80A is insoluble in any of the recommended solvents [25]. To prepare the polymer solution, we used the partial hydrolysis of Pel-80A with hydrofluoric acid. The molecular weight of the resulting polymer was determined from the viscosity of the polymer solutions using characteristic viscosity (the Shultz–Blashke equation) and by gel-exclusion chromatography in the range of 110–130 kDa [25,30].

**Figure 1:**
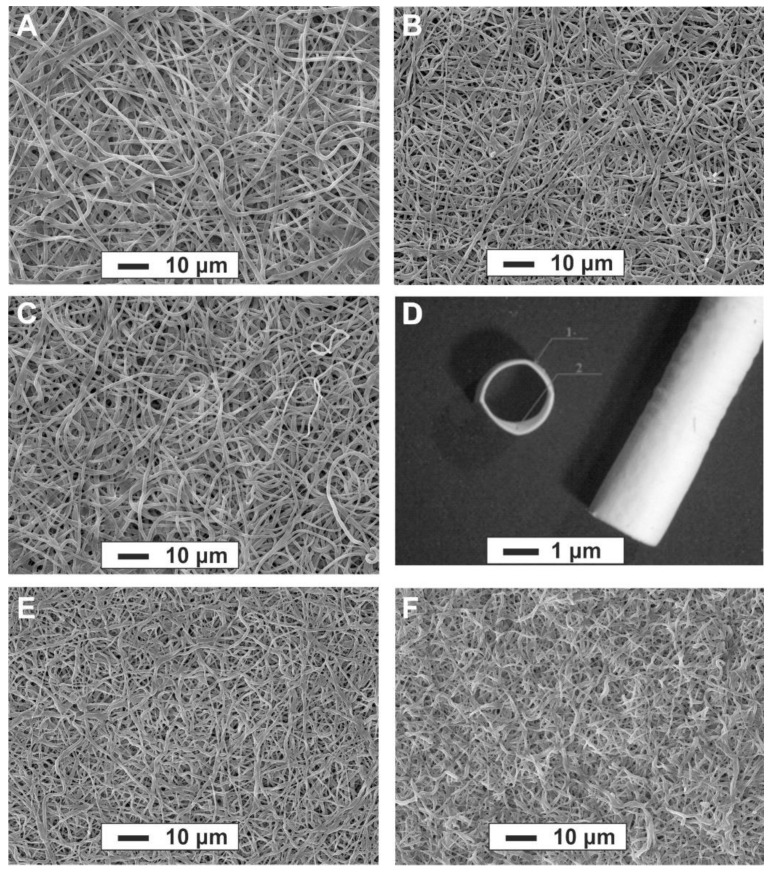
SEM images of electrospun matrices of the following solutions in HFIP: 3.5% Pel-80A scaffold (**A**); 3.5% Pel-80A + 5% Gel scaffold (**B**); 3.5% Pel-80A + 10% Gel scaffold (**C**); Image of electrospun graft containing 3.5% Pel-80A + 10% Gel + 1.5% Bv; scale bar, 1 mm (**D**); 3.5% Pel-80A + 5% Gel scaffold after incubation in PBS for 15 days (**E**) and 30 days (**F**); scale bar, 10 μm; magnification, 1000×.

Electrospinning conditions (concentration of PU and biopolymers, flow rate, voltage, etc.) were optimized to determine the nanofiber morphology of scaffolds using a rotating mandrel for SDVG fabrication. SDVGs were produced 60–75 mm in length with an inner diameter of 1.8 mm and wall thickness of 110 ± 15 µm (Figure 1D). The inner layer of the graft (approximately 10 µm thickness) contained a blend of Pel-80A with the addition of Gel and Bv, while the outer layer contained a blend of Pel-80A with Gel. SEM demonstrated that 3D matrices consist of 1–1.5 µm randomly-oriented fibers with a pore size ranging from ~2.3 to ~3.8 µm, independent of the presence or absence of Gel and Bv (Figure 1A–C).

Using the stress-strain plot (supplementary data), the mechanical properties of the scaffolds were determined and are summarized in Table 1. As is evident from the data in Table 1, GL concentration in an electrospinning solution and hydration of protein in the fiber composition affects the tensile strength of the electrospun materials and their ultimate elongation. We found that materials with the addition of Gel 5–10% had the higher tensile strength in dry and wet conditions. The wetting of matrices decreases their strength in the elastic deformation area by about 20% (compared to dry), and similar properties were expected of SDVGs. There was almost a complete absence of shrinkage of matrices made of Pel-80A, in contrast to matrices made of Tecoflex-80A, which can shrink up to 33% [23]. We found that a one month incubation of Pel-80A–based scaffolds in phosphate buffer does not affect their structural (Figure 1B,E,F) and mechanical properties. The data agree with data published on the stability of PU-based vascular grafts in experiments with animals [27]. It should be noted that the addition of bivalirudin in the electrospun solution did not affect the mechanical properties of the material.

Despite the introduction of gelatin into the blend for electrospinning, the matrix had a 110° contact angle. A similar tendency was observed for the matrices prepared by the electrospun method from solutions of Tecoflex with gelatin [23]. The close interaction between the PU and protein molecules is responsible for this phenomenon [25].

### 3.2. Interaction of Endothelial Cells and Blood with Matrices

**Figure 2:**
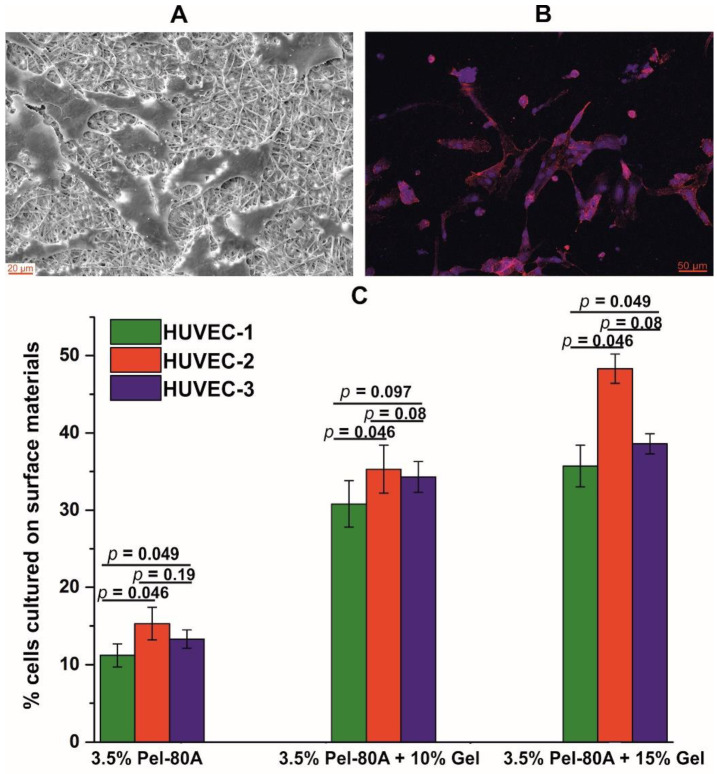
Interaction of endothelial cells with electrospun Pel-80A-based scaffolds. SEM image of endothelial cells cultured on 3.5% Pel-80A + 10% Gel scaffold (**A**). Fluorescence image of cell morphology of HUVEC cultured on the surface of the scaffold containing 3.5% Pel-80A + 15% Gel (nuclei and actin filaments of cells stained with Hoechst 33342 and Phalloidin-TRITC dye, respectively) (**B**). Viability of HUVEC from 3 different donors on the surface of different 3D matrices after 48 h of cultivation (data are presented as the mean of three replicates with standard deviation) (**C**).

SEM and fluorescent microscopy results demonstrated the ability of the HUVEC cells to attach to the surface of the matrices containing Pel-80A with gelatin after 24 h of incubation (Figure 2A,B). The endothelial cells interacted with microfibrous surfaces of matrices and typically had the morphology of HUVEC.

Cell proliferation at the surface of Pel-80A-based materials was assayed using the Alamar Blue reagent versus control cells seeded in the same well-plates on the surface of polystyrene (Figure 2C). The obtained data showed that the addition of gelatin to the electrospun solution leads to at least a two-fold increase in the number of cells adhered to and proliferated on the surface. At that point, similar numbers of HUVEC adhere to the materials containing 10% and 15% of Gel, thus demonstrating the superior performance of 3.5% Pel-80A + 10% Gel matrices with respect to Gel consumption and minimization of allogeneic protein in the implant.

**Figure 3:**
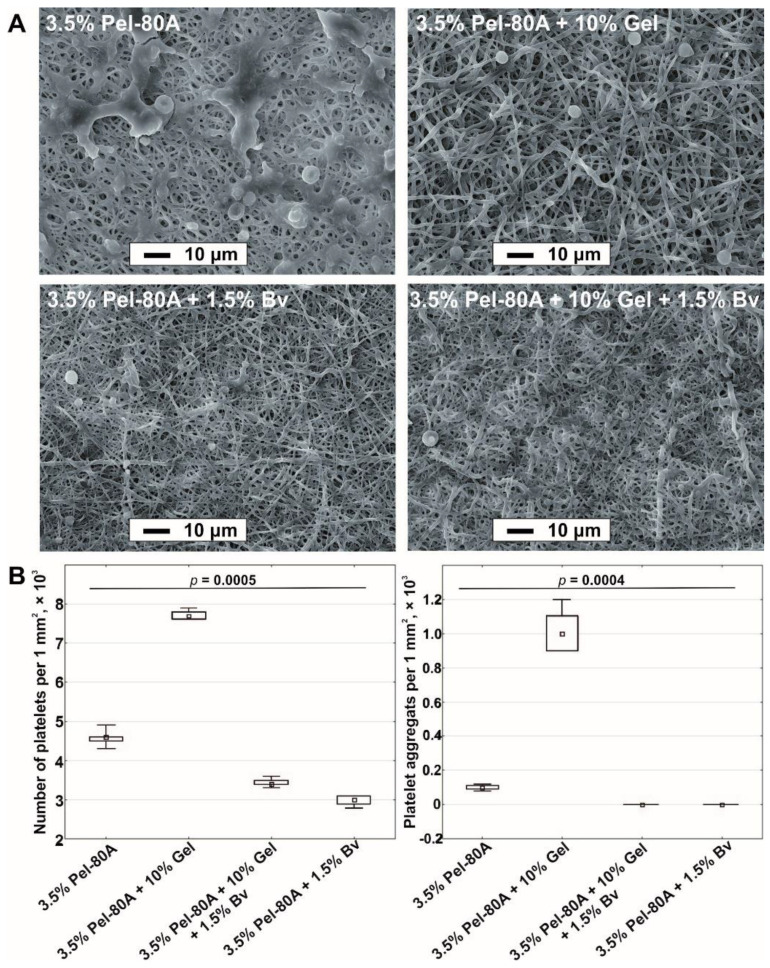
Blood contact test for different scaffolds. SEM images obtained after incubation of electrospun tubes with blood (scale bar, 10 μm; magnification, 1000×) (**A**). Quantitative data of adhered platelets and their aggregates on surface tubes are presented as a median and interquartile range (**B**).

The interaction of blood cells with electrospun matrices was studied using SEM. The obtained data demonstrated that the addition of Bv to the electrospinning solution decreased the binding of platelets and the number of platelet aggregates both on the surface of 3.5% Pel-80A and 3.5% Pel-80A + 10% Gel materials (Figure 3).

The general characteristics of hemostasis influenced by contact of biomaterials—like activated partial thromboplastin time (APTT), D-dimers, and hemolysis—with blood were evaluated and are presented in Table 2.

We found that blood protractedly passed through silicon tubes (diameter 1.8 mm) and blood passed in parallel through grafts produced from 3.5% Pel-80A and 3.5% Pel-80A + 10% Gel had a similar activated partial thromboplastin time. When blood was passed through tubes from 3.5% Pel-80A + 1.5% Bv and 3.5% Pel-80A + 10% Gel + 1.5% Bv, the APTT time increased by 11.3 and 13 times, correspondingly. The concentration of D-dimers did not differ for blood that passed through all tested tubes. The introduction of biopolymers into the fiber led to a two-fold decrease in the level of hemolysis. Tubes produced from 3.5% Pel-80A + 10% Gel + 1.5% Bv demonstrated the lowest hemolysis rate among the electrospun tubes.

### 3.3. In Vivo Study of Pel-80A-Based SDVGs

**Figure 4:**
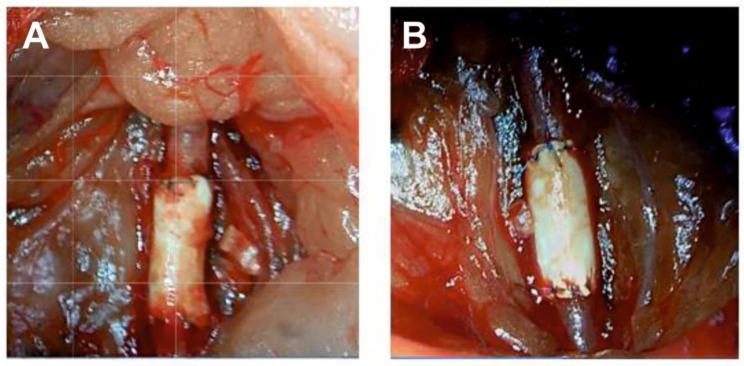
Intraoperative images of ePTFE (**A**) and Pel-80A-based (**B**) vascular grafts during surgical implantation in rats.

Electrospun Pel-80A-based grafts (3.5% Pel-80A + 10% Gel + 1.5% Bv) showed sufficient resistance to surgical intervention during implantation; no edge splitting and good suture retention strength (160 g for polypropylene monofilament suture 8-0) increased convenience during surgery. Impregnation of the wall with blood cells after the start of blood flow was not observed for the electrospun grafts compared to the control ePTFE grafts (Figure 4).

The grafts were implanted in 36 Wistar rats; the results of implantation are summarized in Table 3. Of the implanted VGs, 94.5% of electrospun grafts and 66.7% of the ePTFE grafts (*p* = 0.044) were free of occlusion over the observation period. In the postoperative period, there was no diastasis, suppuration, or other complications of the postoperative wound, surrounding tissues, and the prostheses.

At the first week, ePTFE grafts implantation and acute thrombosis of the SDVG were observed in three cases with the development of acute ischemia of the hind limbs on days 3, 4, and 5. No cases of early thrombosis were observed in animals with implanted Pel-80A-based grafts. At 12 weeks of observation, in two cases, ePTFE implants occlusion of SDVGs occurred with the development of a clinic of acute ischemia of the hind limbs on days 41 and 65. No cases of occlusion PU-based SDVGs were observed. At the 24-week follow-up, there were no lethal outcomes; however, in both groups, according to ultrasound data, one case of prosthesis occlusion was recorded (without the development of clinical of acute ischemia of the hind limbs).

**Figure 5:**
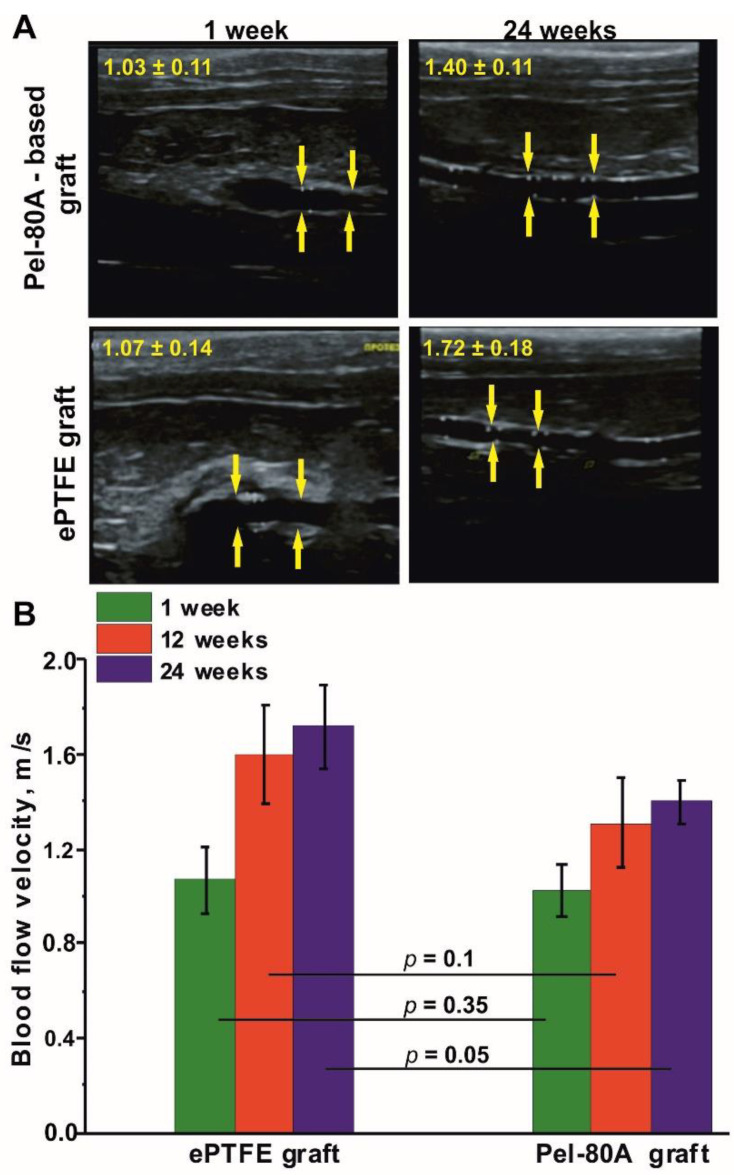
Images from ultrasound examination of vascular grafts (**A**). Blood flow velocity in tested VGs, *n* = 6 (**B**). Blood flow velocity of rat aorta is =1.06 ± 0.13 m/s. Anastomose zones are marked by yellow arrows.

Data obtained by ultrasound examination (Figure 5) demonstrated that the greatest increase in linear blood flow velocity at all times of implantation occurred in the ePTFE group compared with electrospun grafts (1.61 and 1.35 time, correspondingly), indicating a decrease of PTFE VG lumen.

**Figure 6:**
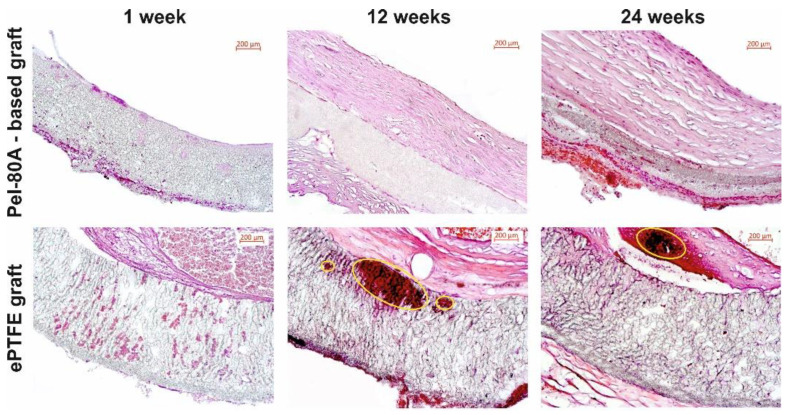
Hematoxylin and eosin images of studied VGs at different times after implantation (lumen of graft appears in the top right). The areas of calcification were indicated by yellow ovals. Scale bar is 200 μm.

The results of the histological examination of the grafts are presented on Figure 6. The inner surface of ePTFE grafts contained thrombotic masses represented by red blood cells and fibrin fibers at one week. In the case of Pel-80A-based grafts, the inner wall volume was free from any thrombotic masses and some lymphocytes were only observed in locations on the external surface of the VG wall. These data confirmed the impermeability of the Pel-80A-based VG for blood cells, as was observed after blood flow start immediately after implantation. No intima layer formed at the surface of the Pel-80A-based VGs.

After three months, the grafts of both types contained a neointimal layer but the neointima in Pel-80A and e-PTFE VGs had distinctive features. The neointima of Pel-80A-based graft presented a homogeneous structure with unidirectional collagen fibers, numerous fibrocytes, and a well-formed monolayer endothelium. The neointimal layer of ePTFE VG had a heterogeneous structure with multidirectional collagen fibers, dissection and calcification areas, and microthromboses on the endothelial surface.

Six months later, the intimal layer of the electrospun VGs contained collagen fibers with a firm structure and numerous fibrocytes. The inner surface was covered by well-formed monolayer endothelium. ePTFE grafts showed diffuse neointimal degeneration with necrosis, calcification, and a thrombosis area (Figure 6).

**Figure 7:**
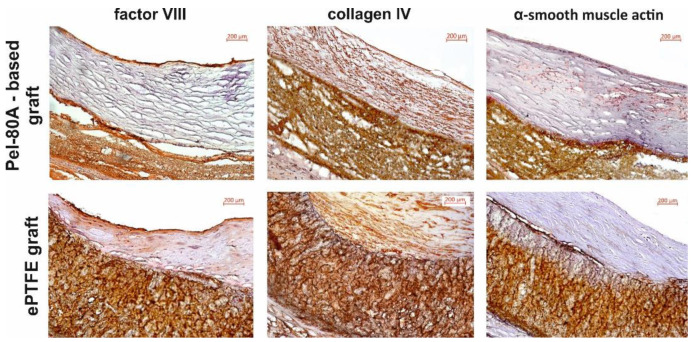
Immunohistochemical staining of VGs at 24 weeks after implantation in rats (lumen of graft appears in the top right). Scale bar is 200 μm.

The presence of the endothelial layer was confirmed by the expression of factor VIII and type 4 collagen within the basement membrane and in the thickness of the formed intima (Figure 7). Expression of α-SMA in the formed neointima was not detected in either type of VG.

In the walls of the studied grafts, we found different levels of calcification. The property of calcification had distinctive features for Pel-80A-based and ePTFE grafts. For electrospun VGs, we found homogeneous and diffuse calcification, whereas for the ePTFE grafts, we observed extensive degeneration areas with coarse lumpy calcification which can lead to injury of graft wall with following inflammation and thrombosis.

The intensity of calcium accumulation depended on the type of prosthesis and the time of implantation of the vascular grafts (Table 4). The ePTFE grafts explanted after 3 and 6 months contained a minimum of three-fold more calcified regions compared with the electrospun grafts implanted for the same period (Table 4).

Intimal layer growth was similar both in ePTFE and Pel-80A-based grafts during first 3 months (Table 4). Thickness of neointima increased by 1.5 times during the second 3 months in ePTFE grafts, whereas further intimal layer growth was not found in Pel-80A-based grafts. This means the e-PTFE VGs accumulate negative changes in the neointima layer with time without a tendency to growth stabilization in contrast to Pel-80A SDVGs.

## 4. Discussion

The literature describes many options for manufacturing SDVGs by electrospinning, using various polymers or their combinations [4,6,7,10,19,21]. Many studies produced significant progress in SDVG tissue engineering by obtaining materials imitating the mechanical behavior of arteries, which are prone to rapid endothelialization, and resistant to thrombosis. However, only a few polyurethane SDVGs are available on the market for hemodialysis access only [31,32,33]. Thus, enhancing the resistance of SVDGs to thrombosis, stenosis, calcification, or infection is still required.

In the present study, we studied electrospun Pellethane-based materials in vitro and in vivo to evaluate their potential for vascular tissue engineering. Previously, electrospun VGs composed of pure Pel-80A polyurethane were shown to have a structure that promotes cell migration into the wall vessel and good biocompatibility when implanted in the aorta of rats [34]. Using aliphatic polyurethanes, the introduction of gelatin into the solution of Tecophilic and Tecoflex for electrospinning increases the mechanical strength of electrospun-produced materials and efficiency of endothelial cell adhesion proliferation [23,26,35,36]. Based on these data, we manufactured and tested Pel-80A-based scaffolds electrospun produced from solutions including polyurethane with gelation or/and bivalirudin.

Polyurethane Pel-80A is insoluble in any of the recommended solvents and their blends, regardless of pretreatment procedures [25,37]. This is probably due to synthesis of Pel-80A in an excess of diisocyanate, and the reaction between terminal isocyanate groups with urethane groups results in allophonate cross-links. Mild hydrolysis with hydrofluoric acid enabled the production of a polymer with molecular weight similar to analogs (Tecoflex EG-80A) that are soluble in HFIP and compatible with Gel and the blends [25]. The produced 3D matrices drastically differed from analogs produced from polyurethane Tecoflex by the presence of two cyclohexyl rings in a hard segment instead of two benzols in Pel-80A PU. Protein-enriched matrices produced from Tecoflex are characterized by a more pronounced dependence of their tensile strength on the composition and wetting of the material [23]. In contrast to Tec-80A-based matrices, which are more elastic compared to Pel-80A analogs, Pel-80A-based matrices are not prone to shrinkage after electrospinning. Shrinkage of Tecoflex-based materials drastically complicates manipulation with such materials (like production of vascular grafts) and Pel-80A-based materials have advantages in this respect.

The mechanical properties of Pel-80A-based materials (Table 1) exceeded those of natural arteries and veins [38,39,40,41]. Elongation at the break of Pel-80A-based matrices only exceeded elongation in elastic deformation area by 1.2 times, whereas strength in this area exceeded the tensile strength of natural carotid arteries by no less than 1.5–2.1 times and showed a similar elastic modulus [35,40]. Pel-80A-based matrices that are stable in phosphate buffer and blood [25] are suitable materials for production of the elements of the cardiovascular system due to their structural, mechanical, and chemical aspects.

To increase biocompatibility, matrices were produced from blends of Pel-80A with Gel, which improves cellular adhesion. We found that HUVEC adhesion and proliferation efficacy weakly depended on Gel concentration in fibers, potentially due to the higher protein concentration in the upper layer of the fibers than its medium concentration [42]. The efficacy of HUVEC binding with surface Pel-based scaffolds was not as high as previously found for Tecoflex-based matrices [23,26] but was two times higher than at the surface of Pel-80A-based materials alone.

The interaction of blood cells with electrospun matrices plays a critical role in the fabrication of materials using vascular grafts [43,44]. Here, we found that the introduction of gelatin in the material reduced hemolysis, but promoted platelet adhesion compared to Pel-80A alone. A similar effect of gelatin introduction on erythrocyte hemolysis was previously observed [35]. To reduce platelets adhesion and increase the hemocompatibility of the materials, a bivalirudin-peptide fragment of the anti-coagulant hirudin was introduced into the electrospun solution along with Gel. Through a triple mechanism of action—inhibition of plasma thrombin, clot bound thrombin, and collagen-induced platelet activation—bivalirudin may act as a more effective reagent for VG wall modification than heparin [45]. The highest hemocompatibility of 3D matrices with respect to clot formation and lysis, hemolysis, and binding of platelets was noted for 3.5% Pel-80A + 10% Gel + 1.5% Bv matrices. Bivalirudin, as previously demonstrated, does not interfere with HUVEC adhesion and proliferation [26]; thus, 3.5% Pel-80A + 10% Gel + 1.5% Bv can be used for production of cardiovascular devices contacting blood.

The in vivo study of electrospun grafts produced from the solution of Pel-80A polyurethane with gelatin and bivalirudin demonstrated potential as an SDVG. Experimental SDVGs with bivalirudin in the inner layer were less prone to thrombosis or occlusions both at early and late follow-up compared to ePTFE grafts (one in the experimental group in the early stages and zero in the late stages versus three in the ePTFE group). Occlusions in the ePTFE group were not associated with defects in surgical technique or infection.

The morphology and microstructure of Pel-80A-based SDVGs prevent the penetration of blood cells into its wall, unlike ePTFE grafts that had walls soaked with blood and filled with blood cells after the blood flow started. The impregnation of the VG wall with blood cells is one of the risk factors for thrombosis and calcification [46,47]. The results of histological examination demonstrated that more pronounced calcification of the SDVG walls was observed for ePTFE SDVGs compared with the electrospun SDVGs. Since the intensity of calcification correlates with the development of local inflammation [48], the more pronounced focal calcification observed in the VG from ePTFE can be interpreted as a negative feature that induces inflammation and stenosis of these grafts. The immunohistochemical study demonstrated that the inner surface of Pel-based SDVG was more densely populated with endotheliocytes compared to ePTFE-based grafts, as evidenced by the circular expression of Factor VIII. The formation of an endothelial monolayer on the surface of the vascular graft prevents thrombus formation and reduces the formation of neointima in the lumen of the vessel in the long-term [6,19,43,49]. The results of immunohistochemical studies are consistent with ultrasound data, which showed the greatest increase in blood flow velocity in the area of ePTFE grafts, confirming the decrease in the effective section of the prosthesis and an increase in the thickness of the neointimal layer compared to the Pel-80A-based SDVG.

The results from the in vivo study showed than electrospun Pel-based grafts demonstrated suitable characteristics compared to the clinically used ePTFE vascular grafts. These data agree with the data obtained by other authors and our previous study, which demonstrate the higher hemocompatibility and biocompatibility of vascular prostheses based on polyurethanes produced via electrospinning in small animal models (mouse and rat) compared to prostheses based on ePTFE [27,50,51,52]. Very close mechanical properties of dry and wet Pel-80A materials and their low shrinkage make Pel-80A SDVG more convenient for production and subsequent manipulations, whereas high Pel-80A stability [25] provides long term functioning after implantation. It should be noted that the model of the rat abdominal aorta is not entirely correct and the data obtained require confirmation in large animals. Anyway, the data indicated that Pel-80A-based SDVG is an appropriate candidate for further studies in preclinical animal models and can find its niche in human SDVG.

## 5. Conclusions

This study described physicochemical properties, biocompatibility, and hemocompatibility of electrospun materials produced from Pel-80A polyurethane blends Pel-80A with gelatin or/and bivalirudin. Pel-80A-based electrospun two-layered grafts and control grafts from ePTFE were compared in an abdominal aortic rat model over 24 weeks. Results of in vivo experiments demonstrated that electrospun VGs showed good vessel remodeling and long-term graft patency compared to ePTFE-grafts. Thus, electrospun grafts prepared from polyurethane with the addition gelatin and bivalirudin are suitable candidates to test in large animals.

## Figures and Tables

**Table 1:** Physical characteristics of Pel-80A-based electrospun matrices.

Matrix Composition, Condition	Tensile Strength (MPa)	Elongation at Break (%)	Elastic Modulus (MPa)	Elastic Strain (%)	Water Contact Angle (°)
3.5% Pel-80A, dry	6.07 ± 0.32	156 ± 19.8	5.67 ± 0.4	107.5 ± 3.5	115.3 ± 0.2
3.5% Pel-80A, wet	5.03 ± 0.69	142 ± 21.3	4.43 ± 0.34	106.5 ± 3.5	-
3.5% Pel-80A + 5% Gel, dry	8.14 ± 1.19	190 ± 27.5	6.81 ± 0.41	155.5 ± 0.7	112.5 ± 1.5
3.5% Pel-80A + 5% Gel, wet	7.86 ± 0.50	195 ± 20.5	5.45 ± 0.38	126.5 ± 4.9	-
3.5% Pel-80A + 10% Gel, dry	8.41 ± 0.48	192 ± 36.2	5.17 ± 0.43	116 ± 4.5	110.2 ± 1.9
3.5% Pel-80A + 10% Gel, wet	6.77 ± 0.46	124 ± 51.0	3.93 ± 0.08	77 ± 1.4	-
3.5% Pel-80A + 15% Gel, dry	6.76 ± 0.29	125 ± 13.5	5.36 ± 0.33	78 ± 1.2	111.7 ± 3.3
3.5% Pel-80A + 15% Gel, wet	6.05 ± 0.56	122 ± 21.0	5.25 ± 0.46	70 ± 1.4	-

**Table 2:** Hemocompatibility of electrospun scaffolds.

Sample	APTT (s)	D-Dimers (µg/mL)	Hemolysis (%)
Control blood	30.4 ± 3.94	0.19 ± 0.02	-
3.5% Pel-80A	30.4 ± 3.66	0.22 ± 0.03	8.51 ± 0.18
3.5% Pel-80A + 10% Gel	30.7 ± 3.44	0.24 ± 0.01	3.14 ± 0.32
3.5% Pel-80A + 1.5% Bv	340.0 ± 20.0	0.22 ± 0.01	4.23 ± 0.47
3.5% Pel-80A + 10% Gel + 1.5% Bv	390.0 ± 10.0	0.22 ± 0.03	3.39 ± 0.69
*p*	0.02	0.18	0.03

**Table 3:** Postoperative complications in the studied groups during the observation period.

Parameter	Group with Pel-80A-Based VGs (*n* = 18)	Group with ePTFE VGs * (*n* = 18)	*p*
Mortality rate, *n* (%)	0 (0)	5 (27.7)	0.045
Graft occlusion, *n* (%)	1 (5.5)	6 (33.3)	0.044
Occlusion to day 7, *n* (%)	0 (0)	3 (16.7)	0.23
Occlusion after day 7, *n* (%)	1 (5.5)	3 (16.7)	0.29

* Reprinted with permission from [27].

**Table 4:** The calcification and the thickness of the neointima in studied VGs at different observation time points.

Studied VGs	Calcification (%)			Thickness of Neointima (µm)		
	3 Months	6 Months	*p* *	3 Months	6 Months	*p* *
Pel-80A based VGs	4.55 ± 0.58	9.75 ± 0.88	0.028	41.84 ± 20.21	40.22 ± 27.71	0.753
ePTFE VGs	6.36 ± 0.56	13.65 ± 1.85	0.027	40.85 ± 22.21	60.76 ± 24.12	0.248
*p*	0.005	0.005	-	0.810	0.378	-

* Horizontal comparison for the parameters within one group at 3 and 6 months of observation.

## Data Availability

Data is contained within the article or supplementary material.

## Supplementary Materials

The following are available online at https://www.mdpi.com/article/10.3390/ma14133678/s1; Figure S1: Typical tensile stress diagrams of electrospun Pel-80A-based scaffolds.
