# Supplementary figures and images for: Assessment of Electrospun Pellethane-Based Scaffolds for Vascular Tissue Engineering
Source: Materials (Basel). 2021 Jul 1;14(13):3678. doi: 10.3390/ma14133678 (PMC8269885; doi:10.3390/ma14133678)

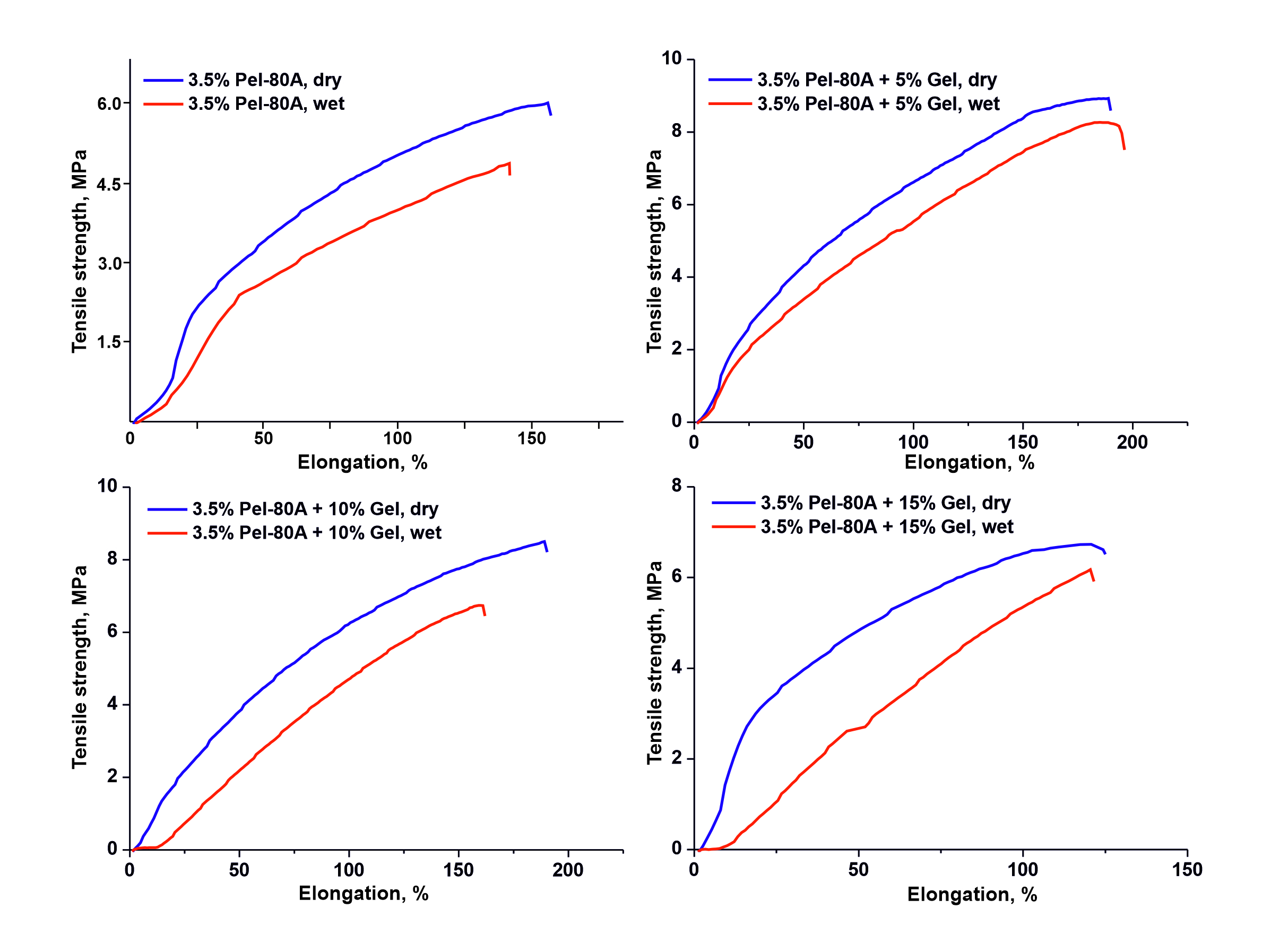

Supplement: Supplementary file 1 [file materials-14-03678-s001.zip › materials-1243633-supplementary.jpg]
